# Differential Analysis of Hemogram Parameters and Cellular Ratios in Severe Asthma Exacerbations: A Comparative Study of Eosinophilic and Non-Eosinophilic Phenotypes

**DOI:** 10.3390/life15060970

**Published:** 2025-06-18

**Authors:** Nicolae Demenciuc, Corina Eugenia Budin, Corina Ureche, Mircea Stoian, Teodora Nicola-Varo, Dragos-Florin Baba, Dariana-Elena Pătrîntașu, Diana Deleanu

**Affiliations:** 1 Doctoral School, George Emil Palade University of Medicine, Pharmacy, Science and Technology of Târgu Mureș, 540142 Târgu Mureș, Romania; demenciuc_nicolae@yahoo.com; 2 Pneumology Department, Mureș Clinical County Hospital, 540098 Târgu Mureș, Romania; dpatrintasu@yahoo.com; 3 Pathophysiology Department, George Emil Palade University of Medicine, Pharmacy, Science and Technology of Târgu Mureș, 540142 Târgu Mureș, Romania; corina.budin@umfst.com; 4 Second Internal Medical Department, George Emil Palade University of Medicine, Pharmacy, Science and Technology of Târgu Mureș, 540142 Târgu Mureș, Romania; teodora.nicola-varo@umfst.ro; 5 Department of Anesthesiology and Intensive Care, George Emil Palade University of Medicine, Pharmacy, Science and Technology of Târgu Mureș, 540142 Târgu Mureș, Romania; mircea.stoian@umfst.ro; 6 Intensive Care Unit, Mures Clinical Country Hospital, Street Gheorghe Marinescu No.1, 540103 Târgu Mureș, Romania; 7 Departmant of Cell and Molecular Biology, George Emil Palade University of Medicine, Pharmacy, Science and Technology of Târgu Mureș, 540142 Târgu Mureș, Romania; dragos-florin.baba@umfst.ro; 8 Emergency Institute for Cardiovascular Diseases and Transplant, 540136 Târgu Mureș, Romania; 9 Department of Internal Medicine, Regional Institute of Gastroenterology (IRGH), 400394 Cluj-Napoca, Romania; deleanudiana@yahoo.com

**Keywords:** asthma, eosinophil, exacerbation, neutrophil-to-lymphocyte ratio, complete blood count

## Abstract

Asthma exacerbations are acute worsening episodes in individuals with bronchial asthma, frequently necessitating emergency hospital care. Early differentiation between eosinophilic (≥150 eosinophils/mm^3^) and non-eosinophilic (<150 eosinophils/mm^3^) subtypes plays a crucial role in treatment decisions and identifying patients eligible for biologic therapies. The ExBA Study explored variations in complete blood count (CBC) parameters and derived cellular ratios—namely the neutrophil-to-lymphocyte (NLR), thrombocyte-to-lymphocyte (TLR), and eosinophil-to-leukocyte ratios (ELR)—in adults hospitalized with severe asthma exacerbations. Ninety patients were enrolled and categorized into eosinophilic (*n* = 38) and non-eosinophilic (*n* = 52) groups. Significant statistical differences were observed in the neutrophil and lymphocyte levels, as well as in all three ratios. ROC analysis highlighted the ELR as the most specific indicator of the eosinophilic phenotype (specificity 100%, AUC 0.938, cut-off 0.003), whereas the NLR and TLR showed stronger associations with the non-eosinophilic group (AUC 0.733 and 0.676). No meaningful differences emerged regarding arterial blood gas levels, length of hospital stay, treatment costs, or mortality. A notable association was found between a personal or family history of atopy and the eosinophilic subtype (*p* = 0.0181). This study underscores the relevance of CBC-based ratios in asthma phenotyping during exacerbation events.

## 1. Introduction

Bronchial asthma is defined as a chronic inflammatory disease associated with bronchial hyperreactivity and obstruction [1]. Asthma exacerbations refer to episodes of disease worsening, characterized by the onset or aggravation of pre-existing symptoms such as a cough, dyspnea, wheezing, and chest tightness [2]. Asthma exacerbations can be triggered by viral or bacterial infections, exposure to aerosolized irritants such as cigarette smoke, and aeroallergen exposure in previously sensitized patients [3,4]. Symptom severity can range from mild to severe, including life-threatening cases that require emergency hospitalization in specialized respiratory medicine units. Severe dyspnea and newly developed respiratory failure may, in some cases, limit ventilatory assessment within the first 24–72 h of admission, and imaging evaluations are often restricted to chest radiography [5]. Thus, complete blood count (CBC) and routine biochemical tests are the most frequently used tools in assessing patients with severe asthma exacerbations [6,7,8].

Moreover, early phenotyping of these patients into T2-high and T2-low subtypes is recommended, especially from the first severe exacerbation [9,10].

The most accessible phenotyping criterion at the time of admission is blood eosinophilia (>150 cells/mm^3^, or 300–400 cells/mm^3^ in some studies), as other biomarkers—such as total IgE, fractional exhaled nitric oxide (FeNO), or interleukin quantification via ELISA methods—are often unavailable or yield results long after patient discharge [11,12]. Eosinophilic asthma phenotypes are typically associated with T2-high inflammation, characterized by elevated eosinophil counts and linked to allergic sensitization and favorable responses to corticosteroids and biologics [10,11,12]. In contrast, non-eosinophilic phenotypes, often T2-low, involve neutrophilic or paucigranulocytic inflammation and have been correlated with reduced responsiveness to standard therapies and different pathophysiological mechanisms, such as bacterial colonization or environmental triggers [13,14,15].

Recent studies have demonstrated that eosinophil counts in both sputum and blood are important predictors of the frequency and duration of asthma exacerbations [13,14,15]. Furthermore, eosinophils have been shown to play a role in airway remodeling by releasing transforming growth factor-beta (TGF-β) and cysteinyl leukotrienes (cysLTs) in patients with seasonal allergic asthma or aspirin-exacerbated respiratory disease [16,17]. Therefore, they contribute significantly to the accelerated and irreversible decline of ventilatory function, particularly forced expiratory volume in one second (FEV1) [18].

Given the involvement of blood cells in the pathophysiological mechanisms of systemic inflammation, numerous studies have analyzed the characteristics of blood cell lineages identified through a CBC, as well as their ratios, across various diseases, including respiratory conditions [19,20]. Alongside lung cancer, pneumonia, chronic obstructive pulmonary disease (COPD), pulmonary tuberculosis, and sepsis, bronchial asthma remains an ongoing research focus regarding CBC analysis, aiming to refine disease phenotyping and assess patient eligibility for biologic therapies [21,22,23,24,25]. Although these parameters have been extensively studied in asthma, limited data exist in the literature regarding their changes during severe asthma exacerbations requiring emergency hospitalization.

Therefore, the primary aim of the ExBA Study (Exacerbated Bronchial Asthma Study) was to evaluate the statistical differences in blood cell lineages and cellular ratios, as determined by the complete blood count, in adult patients with eosinophilic and non-eosinophilic phenotypes during severe exacerbations.

This exploratory study aims to generate preliminary insights that will inform the design of a larger, prospective, multicenter trial.

## 2. Materials and Methods

### 2.1. Study Design

The main objective of this study was to analyze and determine the sensitivity, specificity, and cut-off values of the neutrophil-to-lymphocyte ratio (NLR), thrombocyte-to-lymphocyte ratio (TLR), and eosinophil-to-lymphocyte ratio (ELR) using receiver operating characteristic (ROC) curves in eosinophilic versus non-eosinophilic exacerbated asthma.

Additionally, several secondary objectives were established, including the analysis of arterial blood gas parameters such as pH, HCO_3_, and lactate levels between the two phenotypes. Furthermore, data were collected and analyzed regarding the presence of asthma and respiratory pathology risk factors, including smoking, personal or family history of atopy, and the use of medications associated with atopic conditions (e.g., angioedema)—notably, angiotensin-converting enzyme inhibitors (ACEIs).

Another aim was to assess whether the presence of eosinophilia in these patients influenced the length of hospital stay, hospitalization costs, mortality rate, and decisions regarding the initiation and/or combination of antibiotic therapy.

To achieve these study objectives, we aimed to enroll all patients diagnosed with bronchial asthma who were admitted to the Department of Pulmonology of Mureș County Hospital over a consecutive three-month period (1 January 2024–31 March 2024).

This study was conducted in accordance with the Declaration of Helsinki and was approved by the Research Ethics Committee of Mureș County Hospital (Approval No. 7672/28 May 2024).

### 2.2. Sample Collection Procedure

All blood samples were collected as part of routine clinical care, either in the Emergency Department or within the first four hours of ward admission, using standard venipuncture procedures, and analyzed using the hospital’s central laboratory equipment in accordance with institutional protocols. 

### 2.3. Inclusion and Exclusion Criteria

Inclusion criteria included a confirmed diagnosis of bronchial asthma (using GINA 2024 criteria), emergency hospital admission, age ≥ 18 years, and mandatory CBC analysis performed at the time of admission—either in the Emergency Department or within the first 4 h of ward admission [4].

Exclusion criteria included the presence of other pulmonary pathologies unrelated to asthma (e.g., pulmonary fibrosis, lung cancer, chronic obstructive pulmonary disease (COPD), post-thoracic surgery status, pulmonary tuberculosis, etc.) or systemic conditions associated with eosinophilic inflammation (e.g., eosinophilic esophagitis, eosinophilic granulomatosis with polyangiitis, parasitic infections, etc.).

**Figure 1:**
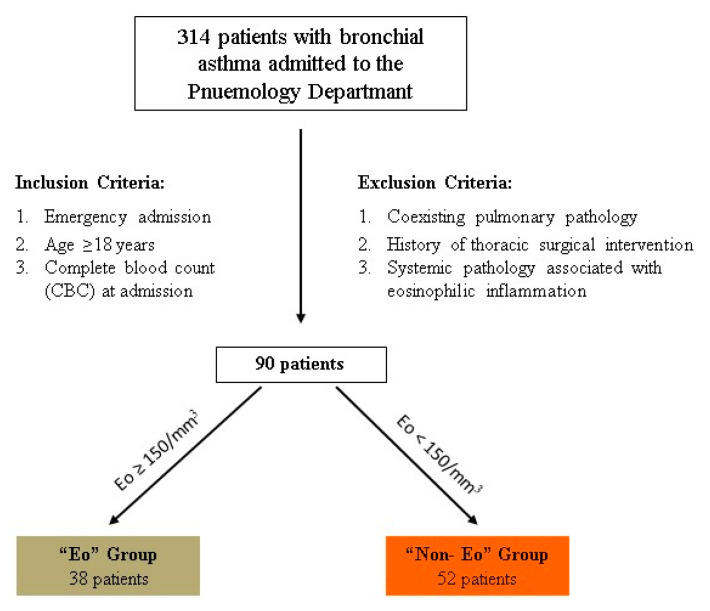
Patient enrollment algorithm in the ExBA Study (inclusion and exclusion criteria).

Following the application of these criteria, out of 314 patients admitted with a diagnosis of bronchial asthma during the three-month study period, only 90 patients remained eligible for inclusion (see Figure 1).

### 2.4. Study Groups

To classify patients into two groups—eosinophilic and non-eosinophilic asthma—the absolute eosinophil count in peripheral blood was used, as measured upon presentation to the Emergency Department at the time of admission or within the first 4 h post-admission. Patients with ≥150 cells/mm^3^ were classified into the eosinophilic group, while those with <150 cells/mm^3^ were assigned to the non-eosinophilic group.

A total of 38 patients were included in the first group (or “Eo” group), defined by ≥150 eosinophils/mm^3^, while the second group, the “Non-Eo” group, was characterized by <150 eosinophils/mm^3^ and included 52 patients.

### 2.5. Statistical Analysis

The data were analyzed using statistical software programs, including Microsoft Excel 2016, GraphPad Prism 10.2.0 (2024), and Epi Info 7.2.6.0 (2023). The statistical tests applied were as follows: the Student’s *t*-test and the Mann–Whitney test for comparing central tendencies, 2 × 2 contingency tables for identifying associations, and ROC curves for determining the sensitivity and specificity of paraclinical tests.

For all tested hypotheses, a 95% confidence interval (CI) was established, and a *p*-value < 0.05 was considered statistically significant.

## 3. Results

In the initial phase, a descriptive analysis of the studied groups was performed. The eosinophilic group included 38 patients, with a mean age of 63 years (SD ± 2 years), comprising 29 women and 9 men. The non-eosinophilic group consisted of 52 patients, with a mean age of 63 years (SD ± 2 years), including 23 women and 29 men.

Subsequently, the central tendencies of complete blood count (CBC) parameters and their derived indices (NLR, TLR, and ELR), arterial blood gas parameters, and selected biochemical markers (pH, HCO_3_, lactate, and effective base excess (EBef)) were compared between the two groups. The obtained data are presented in Table 1 and Table 2.

Statistical analyses revealed a significant difference between the two patient groups regarding the absolute cell count in peripheral blood, as identified through the complete blood count. Specifically, a higher mean neutrophil count and a lower mean lymphocyte count were observed in the non-eosinophilic group compared to the eosinophilic group.

In the case of neutrophils, the mean value was 5808 cells/mm^3^ (SD ± 2825 cells/mm^3^) in the eosinophilic group and 8526 cells/mm^3^ (SD ± 5099 cells/mm^3^) in the non-eosinophilic group, with a statistically significant difference (*p* = 0.0039). However, no significant difference was found between the total white blood cell counts: 9370 cells/mm^3^ (SD ± 2693 cells/mm^3^) in the eosinophilic group and 10,489 cells/mm^3^ (SD ± 5365 cells/mm^3^) in the non-eosinophilic group (*p* = 0.24).

Regarding lymphocytes, the comparison showed a mean value of 2128 cells/mm^3^ (SD ± 1185 cells/mm^3^) in the eosinophilic group and 1357 cells/mm^3^ (SD ± 645 cells/mm^3^) in the non-eosinophilic group, with a significant difference (*p* = 0.0002).

**Figure 2:**
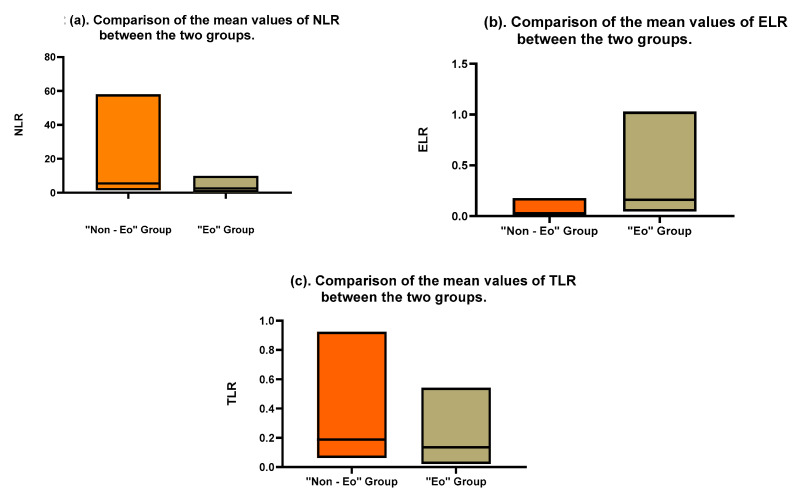
Comparison of the mean values of the NLR (**a**), ELR (**b**), and TLR (**c**) between the two groups.

Additionally, an attempt was made to compare the mean values of the cellular ratios: the NLR, ELR, and TLR. For all three cellular ratios, a statistically significant difference was observed (see Figure 2). The mean value of the NLR for the eosinophilic group was much lower than that for the non-eosinophilic group, at 3.644 cells/mm^3^ (SD ± 2.71 cells/mm^3^) and 9.823 cells/mm^3^ (SD ± 12.19 cells/mm^3^), respectively (*p* = 0.0029).

The mean values of the TLR were 0.1698 cells/mm^3^ (SD ± 0.1086 cells/mm^3^) and 0.2742 cells/mm^3^ (SD ± 0.2183 cells/mm^3^) (*p* = 0.0081), despite the fact that the platelet count did not show a significant difference between the two groups: 277.7 cells/mm^3^ (SD ± 94 cells/mm^3^) and 273.2 cells/mm^3^ (SD ± 91 cells/mm^3^) (*p* = 0.81).

Additionally, the mean value of the ELR was 0.0383 cells/mm^3^ (SD ± 0.0437 cells/mm^3^) for the “Eo” group and 0.2414 cells/mm^3^ (SD ± 0.2269 cells/mm^3^) for the “Non-Eo” group (*p* < 0.0001).

No statistically significant differences were found when comparing certain biochemical parameters (pH, HCO_3_, lactate, and base excess) and arterial blood gas pressures (pO_2_ and pCO_2_), as shown in Table 2.

To achieve the secondary objectives of the ExAB Study, we compared the age at admission, the number of days hospitalized, the number of days spent in the intensive care unit, hospitalization costs, and the in-hospital mortality rate between the two groups, as presented in Table 3. In terms of patient age, length of hospital stay, and hospitalization costs, no statistically significant differences were found between the two groups (*p* = 0.99, *p* = 0.91, and *p* = 0.92, respectively).

On the other hand, we compared the cumulative number of days spent in the intensive care unit for all patients in the “Eo” group, 5 out of 189 days (2.64%), and in the “Non-Eo” group, 10 out of 256 days (3.90%).

Using contingency tables, we assessed the association between gender, smoking, use of angiotensin-converting enzyme inhibitors, and personal or family history of allergy with the eosinophilic phenotype, as shown in Table 4.

Regarding the association between bronchial asthma and sex, a higher prevalence of the condition was observed in females: 52 women (57.7%) and 38 men (42.3%) out of the total number of enrolled patients. However, no statistically significant results were found concerning the association of female sex with the eosinophilic phenotype (*p* = 0.67), and a similar lack of significance was observed for smoking and the use of angiotensin-converting enzyme inhibitors (*p* = 0.384 and *p* = 0.0553, respectively). On the other hand, a personal or family history of atopy or bronchial asthma was found to be associated with the eosinophilic phenotype in the ExAB study (RR = 2.641, CI 1.231–5.663, *p* = 0.0181).

Following statistical analysis regarding the decision to initiate either monotherapy or combined antimicrobial therapy, no significant association was found between the groups, neither in the initiation nor escalation of therapy (*p* = 0.3949 and *p* = 0.3739, respectively), as shown in Table 5.

**Figure 3:**
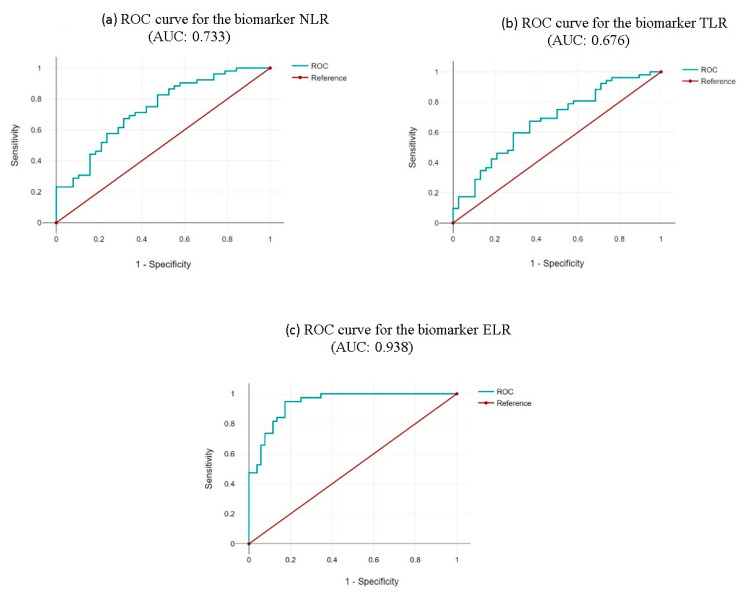
ROC curves for the biomarkers (**a**) NLR, (**b**) TLR, and (**c**) ELR in severe asthma exacerbation.

Referring to the analysis of cell ratios (Table 6), the NLR has a sensitivity of 57.38% and specificity of 58.62% for the “Non-Eo” group (non-eosinophilic phenotype), with an area under the curve (AUC) of 0.733 (*p* = 0.0002), and a determined cut-off value of 4.090, as shown in Figure 3a.

Sensitivity and specificity for the TLR biomarker were then calculated, yielding values of 54.44% and 69.57%, respectively, with an area under the curve (AUC) of 0.676 (*p* = 0.0045) and a cut-off value of 0.1646, for the non-eosinophilic phenotype, as shown in Figure 3b.

Similarly, for the cellular ratios previously analyzed, the ROC curve analysis revealed a sensitivity of 47.50% and a specificity of 100%, with an AUC of 0.938 (*p* = 0.0001) and a cut-off value of 0.003, for the eosinophilic phenotype, as shown in Figure 3c.

### 3.1. Study Limitations

The main limitation of the current study is its retrospective, single-center design, which may affect the generalizability of the findings across different healthcare settings.

Furthermore, the use of the eosinophil count, both as a group-defining criterion and a basis for calculating the ELR, introduces a degree of circularity, potentially inflating the diagnostic performance of the ELR.

Unfortunately, due to the emergency context of patient admission and the severity of exacerbations, spirometry could not be performed upon presentation. Many patients exhibited significant respiratory compromise, making accurate and reproducible spirometry infeasible.

The absence of spirometry data and key biomarkers, such as C-reactive protein (CRP) and interleukins, restricts a more comprehensive phenotypic and endotypic classification of asthma exacerbations.

Although a trend toward differences between the two groups was observed, statistical significance could not be determined due to the limited number of hospitalization days. Additionally, there was a difference in mortality rates (one death per 38 hospitalizations versus one death per 52 hospitalizations). However, similar to the previously analyzed parameter, statistical significance was not demonstrated. Nevertheless, we considered it relevant to mention these parameters, as they may be further evaluated in future studies on larger patient cohorts.

Furthermore, the assessment of blood gas parameters was conducted on a relatively small sample size (12 patients in the “Eo” group and 18 patients in the “Non-Eo” group) due to the unavailability of these tests at the time of admission to the Pulmonology Department.

Additionally, the absence of clinical characteristics, ventilatory functional assessments, and biological markers of systemic inflammation (e.g., C-reactive protein, fibrinogen, ferritin, etc.) limits the ability to establish a comprehensive clustering of phenotypes and endotypes in severe exacerbations of bronchial asthma.

### 3.2. Discussion and Conclusions

Hematological parameters derived from the complete blood count, such as the neutrophil-to-lymphocyte ratio (NLR), thrombocyte-to-lymphocyte ratio (TLR), and eosinophil-to-leukocyte ratio (ELR), have emerged as accessible and cost-effective markers of systemic inflammation. In the context of asthma exacerbations, these indices may offer valuable insights into underlying immune mechanisms and phenotypic differentiation, supplementing clinical evaluation and guiding therapeutic strategies.

The hematological parameters analyzed in this study—the NLR, TLR, and ELR—provide valuable insight into the inflammatory profile of patients with severe asthma exacerbations. The neutrophil-to-lymphocyte ratio (NLR) is a widely recognized marker of systemic inflammation, reflecting the balance between innate and adaptive immune responses, and has been associated with disease severity and prognosis in various inflammatory and infectious conditions. The thrombocyte-to-lymphocyte ratio (TLR), though less extensively studied, integrates platelet activity—a contributor to both inflammation and tissue remodeling—with lymphocyte-mediated immune regulation, offering an additional perspective on the immune dynamics in asthma.

Neutrophilia and lymphopenia are more common in asthmatic patients with a non-eosinophilic phenotype than in those with an eosinophilic one. This finding is confirmed both through the analysis of individual cell lines and the NLR ratio. It is known that the NLR is an independent prognostic factor, with an estimated cut-off value of 3.5 in the general population [26]. Exceeding this threshold is associated with an increased risk of mortality and complications within the first 30 days [27]. Given the similarities in the NLR values between the healthy population and patients in the eosinophilic group, we can conclude that these patients experience, to a lesser degree, the activation of systemic neutrophil-driven inflammatory mechanisms compared to non-eosinophilic patients or those with community-acquired pneumonia, or other infectious or inflammatory diseases [22,28]. This distinguishes them from the non-eosinophilic phenotype, where 35 out of 52 patients (67.30%) had NLR values exceeding the cut-off of 4, compared to 13 out of 38 patients (34.21%) in the eosinophilic group.

Although no significant difference was observed in the mean platelet count between the two groups, the TLR value was significantly lower in the “Eo” group due to the pronounced lymphopenia observed in the non-eosinophilic group. Therefore, we suppose that in eosinophilic patients, there was no loss of lymphocytic functions such as immune regulation, modulation, immune memory, or cytotoxic capability. However, further, more specific, and detailed preclinical research should be conducted to identify the exact effects of lymphopenia in non-eosinophilic asthmatic patients.

The relevance of the ELR analysis in our study model remains limited, considering that the eosinophil count was an inclusion criterion. However, we deemed it important to report this parameter for future studies as a potential reference marker. Respectively, in our patients, a rather low specificity of this parameter was demonstrated for the eosinophilic phenotype, only 47.50%, which shows that the lymphocyte count response is also very important and should be taken into consideration in future studies regarding the phenotyping of asthmatic patients.

Although non-eosinophilic exacerbations are potentially caused by bacterial infections—suggesting potential alterations in pH and lactate levels—no statistically significant differences were found between the analyzed groups. Similar results were observed in the analysis of arterial blood gas parameters (pO_2_ and pCO_2_).

Risk factors associated with eosinophilic asthma exacerbations include female sex and a personal or family history of atopy or bronchial asthma. Additionally, the association between ACE inhibitor use and the eosinophilic phenotype approached statistical significance, suggesting that future studies with larger patient cohorts, along with the identification and analysis of other phenotypes and endotypes, may highlight a potential link [29].

Given that inflammatory processes in bronchial asthma are dynamic, interrelated, and triggered by various infectious and allergic factors, phenotyping during exacerbations is imperative. A more comprehensive understanding of clinical and laboratory characteristics, by accumulating further data on severe exacerbations of both T2-high and T2-low asthma and their subgroups, would provide better insight into the underlying pathophysiological mechanisms. This, in turn, would guide therapeutic decision-making in clinical practice, enhancing accuracy and precision in the management of asthmatic patients during acute exacerbations.

As an exploratory investigation, this study provides foundational data to guide future large-scale, prospective, multicenter research. The findings serve as a preliminary framework for more comprehensive studies aimed at refining asthma phenotyping during exacerbations.

Future research should address all limitations of this study by implementing a prospective multicenter study design involving a more diverse patient population.

## Figures and Tables

**Table 1:** Mean values of the complete blood count (CBC) parameters in both groups, along with the *p*-value, indicate the significance of the differences between them.

Parameter	Normal Range	Eosinophilic Group	Non-Eosinophilic Group	*p*-Value
Leukocytes (cells/mm^3^)	4000–11,000	9370 (±SD: 2693)	10,489 (±SD: 5365)	0.24
Neutrophils (cells/mm^3^)	1800–7500	5808 (±SD: 2825)	8526 (±SD: 5099)	0.0039
Lymphocytes (cells/mm^3^)	1000–4800	2128 (±SD: 1185)	1357 (±SD: 645)	0.0002
Platelets (cells/mm^3^)	150–400,000	277.7 (±SD: 94)	273.2 (±SD: 91)	0.81
Neutrophil-to-Lymphocyte Ratio (NLR)	1.0–3.5	3.644 (±SD: 2.71)	9.823 (±SD: 12.19)	0.0029
Thrombocyte-to-Lymphocyte Ratio (TLR)	0.1–0.3	0.1698 (±SD: 0.1086)	0.2742 (±SD: 0.2183)	0.0081
Eosinophil-to-Leukocyte Ratio (ELR)	0.01–0.06	0.0383 (±SD: 0.0437)	0.2414 (±SD: 0.2269)	<0.0001

**Table 2:** Mean values of laboratory parameters in both groups, along with the *p*-value, indicate the significance of the differences between them.

Parameter	Normal Range	Eosinophilic Group	Non-Eosinophilic Group	*p*-Value
pH	7.35–7.45	7.39 (±SD: 0.06)	7.421 (±SD: 0.09)	0.42
pCO_2_ * (mmHg)	35–45	37.98 (±SD: 17.38)	38.49 (±SD: 21.54)	0.88
pO_2_ ** (mmHg)	75–100	82.89 (±SD: 42.23)	91.18 (±SD: 31.88)	0.71
HCO_3_ *** (mEq/L)	22–26	23.98 (±SD: 10.91)	25.00 (±SD: 12.32)	0.50
BEecf **** (mEq/L)	−2 to +2	−0.75 (±SD: 3.98)	0.075 (±SD: 4.14)	0.57
Lactate (mg/dL)	4.5–19.8	1.83 (±SD: 1.78)	2.977 (±SD: 0.61)	0.24

* pCO_2_ (partial pressure of carbon dioxide), ** pO_2_ (partial pressure of oxygen), *** HCO_3_ (bicarbonate level in blood), and **** BEecf (base excess of extracellular fluid).

**Table 3:** Mean values of age, length of hospital stay, days spent in the intensive care unit, hospitalization costs, and in-hospital mortality rate between the two groups, along with the *p*-value, indicate the significance of the differences between them.

Parameter	Eosinophilic Group	Non-Eosinophilic Group	*p*-Value
Age (years)	63 (±SD: 2)	63 (±SD: 2)	0.99
Length of hospital stay (days)	4.97 (±SD: 1.74)	4.92 (±SD: 2.63)	0.91
Length of stay in ICU * (days)	5/189 (2.64%)	10/256 (3.90%)	-
Reimbursement value (euros)	EUR 556 (±SD: EUR 626)	EUR 571 (±SD: EUR 907)	0.92
Death (number)	1/38 (2.53%)	1/52 (1.92%)	-

* Intensive care unit.

**Table 4:** Association between sex, smoking, use of angiotensin-converting enzyme inhibitors, and personal or family history of allergy or bronchial asthma and the eosinophilic phenotype.

Parameter	Eosinophilic Group	Non-Eosinophilic Group	Relative Risk (RR)	Confidence Interval (CI)	*p*-Value
Gender	F *—23 M **—15	F—29 M—23	1.121	0.6808–1.844	0.67
Active smoking	Smokers—16 Non-smokers—22	Smokers—17 Non-smokers—35	0.839	0.5681–1.239	0.384
ACE ***	With ACE—22 Without ACE—15	With ACE—19 Without ACE—33	0.6881	0.4691–1.009	0.0553
Personal or family history of allergy (atopy)	With personal/family history of atopy—13 Without a personal/family history of atopy—19	With personal/family history of atopy—8 Without personal/family history of atopy—44	2.641	1.231–5.663	0.0181

* F—female partcipants, ** M—male participants, *** ACE—Angiotensin-converting enzyme.

**Table 5:** Association between the eosinophilic phenotype and the decision to initiate antimicrobial therapy.

Parameter	Eosinophilic Group	Non-Eosinophilic Group	Relative Risk (RR)	Confidence Interval (CI)	*p*-Value
Initiation of antibiotic therapy	With ABX—1 Without ABX—37	With ABX—5 Without ABX—47	0.3949	0.0333–2.249	0.3949
Combined antibiotic therapy	No—25 Yes—12	No—27 Yes—20	1.176	0.8436–1.640	0.3739

**Table 6:** Summary of sensitivity and specificity values for the cellular ratios NLR, TLR, and ELR in exacerbated bronchial asthma in adults.

Neutrophil-to-Lymphocyte Ratio (NLR)	Thrombocyte-to-Lymphocyte Ratio (TLR)	Eosinophil-to-Leukocyte Ratio (ELR)
Sensitivity: 57.38% (non-eosinophilic phenotype) Specificity: 58.62% AUC (Area Under the Curve): 0.733 Cut-off value: 4.090 *p*-value: 0.0002	Sensitivity: 54.44% (non-eosinophilic phenotype) Specificity: 69.57% AUC (Area Under the Curve): 0.676 Cut-off value: 0.1646 *p*-value: 0.0045	Sensitivity: 47.50% (eosinophilic phenotype) Specificity: 100% AUC (Area Under the Curve): 0.938 Cut-off value: 0.003 *p*-value: 0.0001

## Data Availability

The original contributions presented in the study are included in the article, further inquiries can be directed to the corresponding author.

## Abbreviations

ACE	Angiotensin-Converting Enzyme
ACEIs	Angiotensin-Converting Enzyme Inhibitors
AUC	Area Under the Curve
BEecf	Base Excess of Extracellular Fluid
CBC	Complete Blood Count
COPD	Chronic Obstructive Pulmonary Disease
cysLTs	Cysteinyl Leukotrienes
EBef	Effective Base Excess
ELISA	Enzyme-Linked Immunosorbent Assay
ELR	Eosinophil-to-Leukocyte Ratio
Eo	Eosinophilic Group
ExBA	Exacerbated Bronchial Asthma
FeNO	Fractional Exhaled Nitric Oxide
FEV1	Forced Expiratory Volume in One Second
GINA	Global Initiative for Asthma
ICU	Intensive Care Unit
IgE	Immunoglobulin E
NLR	Neutrophil-to-Lymphocyte Ratio
Non-Eo	Non-Eosinophilic Group
pCO_2_	Partial Pressure of Carbon Dioxide
pO_2_	Partial Pressure of Oxygen
RR	Relative Risk
ROC	Receiver Operating Characteristic
SD	Standard Deviation
T2-high	Type 2 Inflammation High
T2-low	Type 2 Inflammation Low
TGF-β	Transforming Growth Factor Beta
TLR	Thrombocyte-to-Lymphocyte Ratio
